# Inhibition of HMGCoA reductase by simvastatin protects mice from injurious mechanical ventilation

**DOI:** 10.1186/s12931-015-0173-y

**Published:** 2015-02-14

**Authors:** Nikolaos Manitsopoulos, Stylianos E Orfanos, Anastasia Kotanidou, Ioanna Nikitopoulou, Ilias Siempos, Christina Magkou, Ioanna Dimopoulou, Spyros G Zakynthinos, Apostolos Armaganidis, Nikolaos A Maniatis

**Affiliations:** 1st Department of Critical Care Medicine and Pulmonary Services, GP Livanos and M Simou Laboratories, University of Athens Medical School, Evangelismos Hospital, Athens, Greece; 2nd Department of Critical Care, University of Athens Medical School, Attikon Hospital, Haidari, Greece; Department of Pathology, Evangelismos Hospital, Athens, Greece

**Keywords:** Ventilator lung injury, Acute respiratory distress syndrome, Acute lung injury, Pulmonary edema, Statin, Lung function, Lung compliance, Endothelial permeability

## Abstract

**Background:**

Mortality from severe acute respiratory distress syndrome exceeds 40% and there is no available pharmacologic treatment. Mechanical ventilation contributes to lung dysfunction and mortality by causing ventilator-induced lung injury. We explored the utility of simvastatin in a mouse model of severe ventilator-induced lung injury.

**Methods:**

Male C57BL6 mice (n = 7/group) were pretreated with simvastatin or saline and received protective (8 mL/kg) or injurious (25 mL/kg) ventilation for four hours. Three doses of simvastatin (20 mg/kg) or saline were injected intraperitoneally on days −2, −1 and 0 of the experiment. Lung mechanics, (respiratory system elastance, tissue damping and airway resistance), were evaluated by forced oscillation technique, while respiratory system compliance was measured with quasi-static pressure-volume curves. A pathologist blinded to treatment allocation scored hematoxylin-eosin-stained lung sections for the presence of lung injury. Pulmonary endothelial dysfunction was ascertained by bronchoalveolar lavage protein content and lung tissue expression of endothelial junctional protein Vascular Endothelial cadherin by immunoblotting. To assess the inflammatory response in the lung, we determined bronchoalveolar lavage fluid total cell content and neutrophil fraction by microscopy and staining in addition to Matrix-Metalloprotease-9 by ELISA. For the systemic response, we obtained plasma levels of Tumor Necrosis Factor-α, Interleukin-6 and Matrix-Metalloprotease-9 by ELISA. Statistical hypothesis testing was undertaken using one-way analysis of variance and Tukey’s post hoc tests.

**Results:**

Ventilation with high tidal volume (HVt) resulted in significantly increased lung elastance by 3-fold and decreased lung compliance by 45% compared to low tidal volume (LVt) but simvastatin abrogated lung mechanical alterations of HVt. Histologic lung injury score increased four-fold by HVt but not in simvastatin-pretreated mice. Lavage pleocytosis and neutrophilia were induced by HVt but were significantly attenuated by simvastatin. Microvascular protein permeability increase 20-fold by injurious ventilation but only 4-fold with simvastatin. There was a 3-fold increase in plasma Tumor Necrosis Factor-α, a 7-fold increase in plasma Interleukin-6 and a 20-fold increase in lavage fluid Matrix-Metalloprotease-9 by HVt but simvastatin reduced these levels to control. Lung tissue vascular endothelial cadherin expression was significantly reduced by injurious ventilation but remained preserved by simvastatin.

**Conclusion:**

High-dose simvastatin prevents experimental hyperinflation lung injury by angioprotective and anti-inflammatory effects.

## Background

Non-cardiogenic pulmonary edema, known as Acute Respiratory Distress Syndrome (ARDS), is a frequent cause of respiratory failure [1]. There are multiple etiologies but the final result is airspace flooding with protein-rich edema due to a leaky and inflamed alveolo-capillary membrane. Mechanical ventilation may exacerbate edema and inflammation by alveolar over-distention (ventilator-induced lung injury -VILI) and, consequently, ventilation with low tidal volume (Vt) is now routinely used [2]. Despite optimal ventilation, several patients with severe lung injury will remain with high inflation pressures and poor oxygenation, facing a mortality risk in excess of 40% [3].

In searching for promising medical therapies for ARDS, we previously showed that pre-treatment with atorvastatin, a 3-hydroxy-3-methylglutaryl-coenzyme A reductase inhibitor (“statin”) can abrogate VILI in isolated rabbit lungs [4]. Given the limited significance of immune cells in this model, the atorvastatin effect likely comprised pre-conditioning of resident lung cells to withstand stretch injury, rather than modifying the immune response. Even though the drug almost completely eliminated lung injury in the *ex vivo* model, its effect cannot be extrapolated in the intact animal. In this work, we expand upon this observation using an *in vivo* mouse model of aggressive VILI with high-dose simvastatin pre-treatment. Statins attract research interest for this indication due to their pleiotropic immunomodulating and endothelial-protective properties [5]. Effects specific to the endothelium, which may help preserve the endothelial barrier in the setting of inflammation, include modulating cytoskeletal rearrangement events, attenuating oxidative burst and improving nitric oxide bioavailability [5]. Accordingly, there is a number of reports demonstrating clear benefit in pre-clinical lung injury elicited by endotoxin, radiation, and mechanical ventilation [6-8], while a partial effect was seen in bacterial pneumonia models [9-11] and no effect was seen in viral pneumonia models [12,13].

In translational studies, simvastatin given to patients with ARDS did not improve mortality, but had a significant effect on extrapulmonary organ dysfunction and a trend towards improvement of pulmonary outcomes [14]. In contrast, in patients with ventilator-associated pneumonia, who were overwhelmingly on statins prior to ICU admission, Papazian *et al*. found no improvement in survival or other relevant outcomes [15]. In a more recent trial in sepsis patients with ARDS, rosuvastatin treatment early into the disease process did not alter any of the clinical outcomes studied [16]. However, relatively few of these patients had severe ARDS [3], which is associated with the highest mortality. Thus, an important subpopulation of ARDS patients may have been understudied. Even more recently, McAuley *et al*. administered simvastatin to a patient population of moderate to severe ARDS but again a clinical impact failed to be demonstrated [17].

Mechanical ventilation is almost universally applied as a means of support for patients with ARDS but can provoke further lung dysfunction. The predominant mechanism is alveolar over-distention (*volutrauma*) [2], which elicits an acute response with local and systemic impact [2]. In ARDS patients this may occur despite use of low Vt, due to preexisting airspace collapse from edema and atelectasis. To elicit VILI in healthy mice, however, ventilation with supra-normal Vt is required. We have previously found that ventilation with a Vt of 25 mL/Kg for four hours causes robust increase in lung tissue elastance coefficient, in parallel with pulmonary microvascular permeability [18,19]. We now hypothesize that pre-treatment with simvastatin *in vivo* preserves pulmonary vascular integrity and lung mechanics in response to tidal stretch. Using a high Vt ventilation protocol we elicited lung injury and measured lung function in addition to morphological and biochemical parameters of experimental lung injury to ascertain possible effects of simvastatin.

## Methods

### Animals

C57BL/6 mice were bred and maintained in the animal facilities of the “Hellenic Pasteur Institute” (Athens, Greece) under specific pathogen–free conditions. Mice were housed at 20–22°C, with 55 ± 5% humidity, a 12-hour light–dark cycle and unrestricted access to food and water. All experimentation was approved by the Evangelismos hospital Research Review Board as well as by the Veterinary Service of the governmental prefecture of Attica. Daily Simvastatin (20 mg/kg, Sigma) or normal saline (NS) intraperitoneal (ip) administration started 2 days prior to the experiment with the final dose applied 1 hour prior to mechanical ventilation. Simvastatin was dissolved in pure ethanol and the stock solution was diluted in NS to a mean volume of 50 μL per injection.

### Experimental design

Mice were randomly assigned to four groups, each comprising 7 animals (n = 7). Control groups were pre-treated with simvastatin or NS and were ventilated with ambient air at low tidal volume (LVt) of 8 mL/kg and respiratory rate of 150 breaths/min. Injury groups were pre-treated with simvastatin or NS and were ventilated with ambient air using high tidal volume (HVt) of 25 mL/kg and respiratory rate of 50 breaths/min.

### Mechanical ventilation

Anesthesia was induced by ketamine/xylazine (100 mg/Kg, 10 mg/Kg respectively) injected ip 1 hour after the final simvastatin or NS administration and maintained by administering 1/3 of the initial dose every 45 min. The trachea was exposed under sterile conditions, cannulated with a 22-gauge catheter and sutured. Mechanical Ventilation was performed using a small animal ventilator (Flexivent, Scireq, Ontario, Canada) and ambient air. Positive end-expiratory pressure was set at 2 cmH_2_O and Vt was set at 8 mL/kg. After an initial 5 min run-in period, two deep inflations to total lung capacity were applied in order to standardize lung volume history, followed by a 3-minute LVt ventilation period at the end of which baseline measurements of lung function were obtained. We then proceeded to LVt or HVt ventilation according to experimental design. Respiratory mechanics were evaluated by measuring tissue elastance coefficient (H) and tissue damping coefficient (G) via forced oscillation technique. Five successive measurements were obtained in 30- second intervals. Following these maneuvers, a single quasi-static pressure-volume curve was transduced in order to measure the compliance (Cst) of the respiratory system [18].

### Samples

Following mechanical ventilation and lung function measurement, mice were sacrificed by exsanguination under deep anesthesia after drawing 400-500 μL of venous blood from the inferior vena cava in heparinized 27 g syringe. Plasma was separated by centrifugation at 1500x*g* for 15 min at 4°C and stored at −80°C. Bronchoalveolar lavage fluid (BALF) was sampled by injecting and slowly withdrawing 1 mL of phosphate-buffered saline (PBS) through the tracheal cannula three times. The three aliquots were pooled, centrifuged at 1500×*g* for 5 minutes at 4°C and supernatants were stored at −80°C, while cell pellets were reconstituted in 1 mL PBS for cell quantification and preparation of cytocentrifugation slides.

The lungs were exposed by a midthoracotomy incision and the pulmonary arteries were perfused with PBS. The right lung was ligated at the right hilum, resected, rinsed in PBS, flash-frozen in liquid nitrogen and stored at −80°C until further analysis. The left lung was inflated with 4% neutral buffered paraformaldehyde instilled at 25 cm H_2_O pressure through the trachea for 120 min. The trachea was tied and the lung was immersed in 4% buffered paraformaldehyde for 24 hours before a graded alcohol dehydration procedure and embedding in paraffin.

### BALF total protein

Total protein concentration in the BALF was determined with the Bio-Rad Dc Protein Assay kit (Bio-Rad Laboratories, Hercules, CA, USA).

### Lung tissue histology

4-μm sections of paraffin-embedded tissues were prepared using a microtome (Leica RM2145). Hematoxylin/eosin-stained lung slides were evaluated by a histopathologist blinded to treatment allocation using a scale from 0 to 4 to determine the degree of interstitial inflammation, alveolar inflammation and alveolar septal congestion [20]. Four non-sequential sections were evaluated.

### BALF total cell count and differentiation

Total cell counting in the BALF was performed manually using an improved *Neubauer* hemocytometer according to standard procedure. To determine BALF cell type, 50x10^3^ cells were plated and spun on glass slides by cytocentrifugation at 500x*g* for 6 min, air-dried and stained according to May-Gruenwald-Giemsa. Differential cell count was performed using an optical microscope (OLYMPUS BX50).

### SDS-polyacrylamide Gel electrophoresis (PAGE)

SDS-PAGE was performed using 12% polyacrylamide slab gels on a Biorad Mini Protean II electrophoresis apparatus (Bio-Rad, Hercules, CA, USA). Electrophoresis was carried out at 120 V for 90 min at ambient temperature. Following electrophoresis, samples were transferred onto an Immobilon-P PVDF membrane (Millipore, 0.45 μl pore size, Millipore Corporation, Billerica, MA, USA). Western transfer was performed on a wet transfer apparatus (Bio-Rad, Hercules, CA, USA). Immunological detection of Vascular Endothelial (VE)-cadherin and β-tubulin was performed with antibodies purchased from Santa Cruz Biotechnology Inc. (Santa Cruz, CA, USA). The final results visualized by enhanced chemiluminescence (PerkinElmer, Inc., Waltham, MA, USA) were developed on Agfa Ortho CP-G Plus films (Agfa HealthCare NV, Mortsel, Belgium). The density of each band was expressed in arbitrary units using β-tubulin as reference.

### Enzyme-linked immunosorbent assay (ELISA)

Tumor Necrosis Factor-α (TNF-α), interleukin-6 (IL-6) and matrix metalloproteinase-9 (MMP-9) were measured in plasma or BALF using ELISA kit (R&D Systems, Inc., Minneapolis, USA) according to the manufacturers’ instructions.

### Statistical analysis

Data are presented as mean (±SEM). Statistical hypothesis testing was undertaken by one- or two-way ANOVA followed by Bonferonni’s or Tukey’s post hoc tests respectively (Graph- Pad Prism, Graph-Pad Software, San Diego, Calif;). p < 0.05 was considered significant.

## Results

### Simvastatin preserves respiratory function in VILI

**Figure 1:**
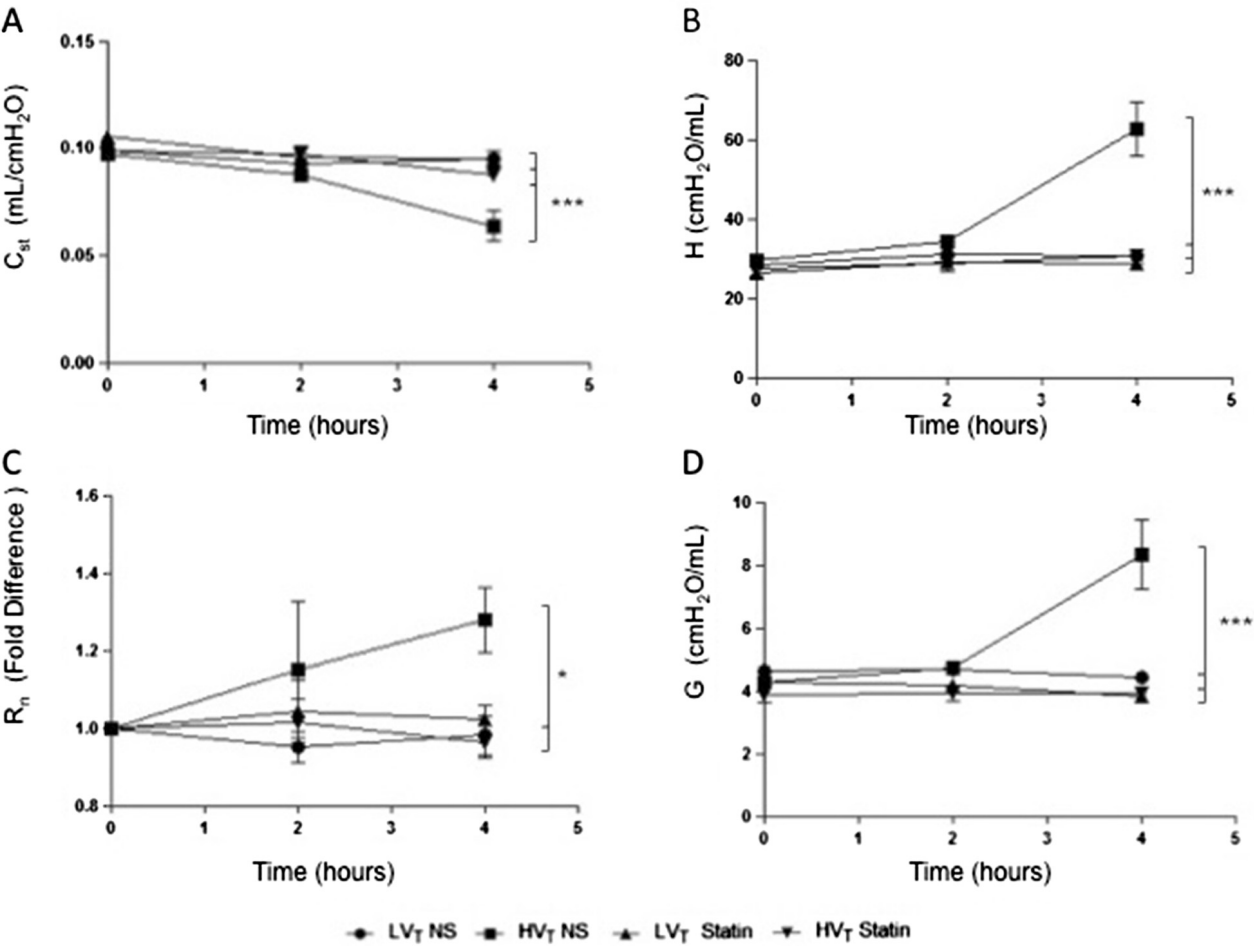
**Lung mechanics in ventilator injury.** Time-course of lung mechanical alterations in mice undergoing ventilation with high (25 mL/Kg) or low (8 mL/Kg) tidal volume for four hours with simvastatin or saline pre-treatment. Static Compliance C_st_ was evaluated by a single quasi-static pressure-volume curve **(A)** while Lung Tissue Elastance coefficient H **(B)**, Airway Resistance R_n_ **(C)**, and Lung Tissue Impedance coefficient G **(D)** were evaluated via forced oscillation technique. (NS: normal saline; Simva: Simvastatin; *denotes p < 0.05; **p < 0.01 and ***p < 0.001).

Decreased lung compliance leading to increased work of breathing [21] can provoke respiratory failure in ARDS. Using a high-stretch ventilation protocol, we observed time-dependent lung mechanics alterations starting to trend at the 2 hr time-point and reaching significance at 4 hours, while no significant changes to baseline were recorded with LVt ventilation (Figure 1). Specifically, elastance coefficient H peaked at 62.69±10.36 cmH_2_O/mL with baseline values at 31.24±0.91 cmH_2_O/mL (Figure 1B), while tissue damping coefficient increased by 1.87-fold at 8.34±1.2 cmH_2_O/mL compared to baseline (Figure 1D). To corroborate the above, quasi-static lung compliance, the reciprocal of elastance, was measured by pressure-volume curve and was reduced by approx. 45% in the HVt group compared to LVt ventilation (Figure 1A). In addition, we recorded a mild but measurable increase in Newtonian airway resistance in the HVt group compared to LVt (Figure 1C). Pre-treatment with simvastatin completely abrogated all mechanical derangements and preserved lung function to the levels recorded at baseline (Figure 1).

### Histological alterations in ventilated lungs are prevented by simvastatin

**Figure 2:**
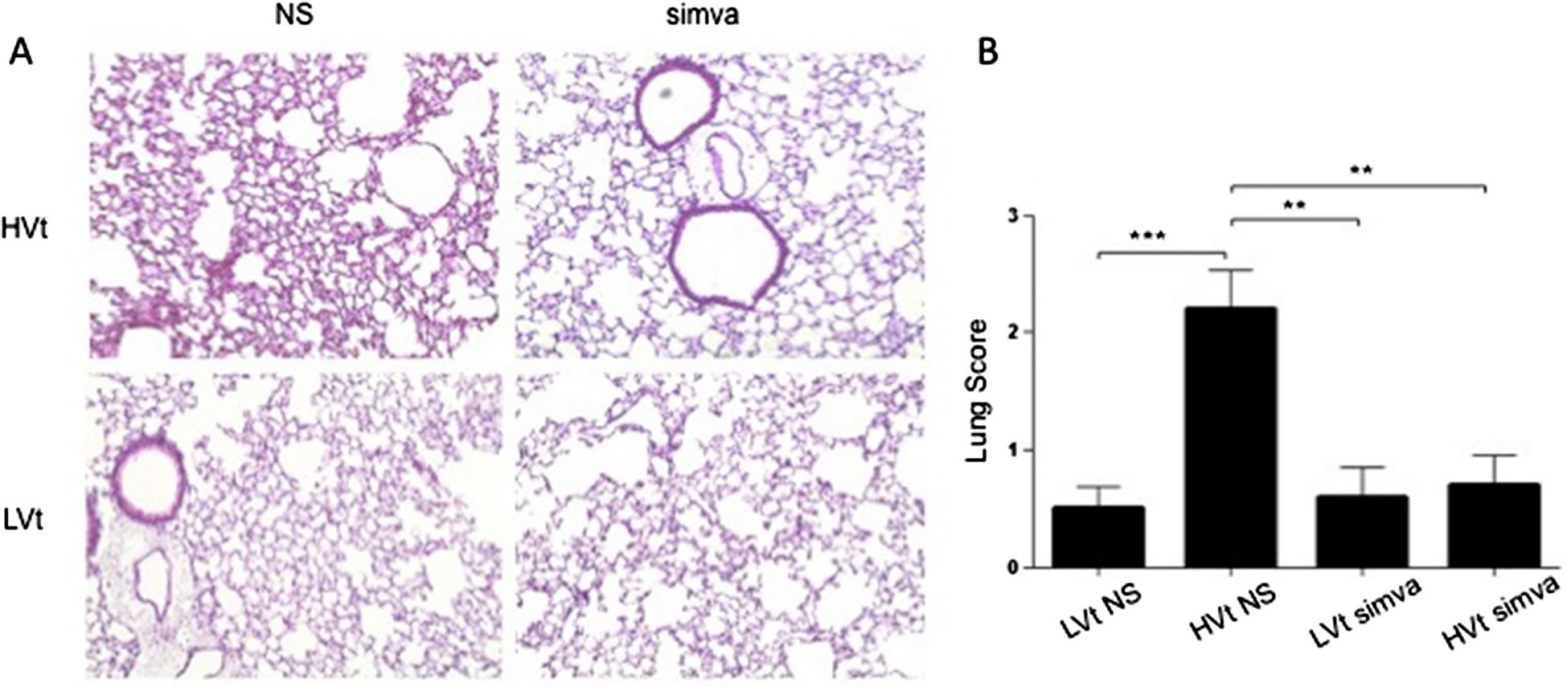
**Lung tissue histology. (A)**. Representative hematoxylin-eosin-stained mouse lung sections following high or low tidal volume (Vt) ventilation with simvastatin or normal saline. **(B)** Semiquantitative lung injury score comprising interstitial inflammation, alveolar inflammation and alveolar septal congestion [20]. (NS: normal saline; Simva: Simvastatin; *denotes p < 0.05; **p < 0.01 and ***p < 0.001).

We evaluated pulmonary effects of mechanical ventilation on hematoxylin-eosin-stained lung sections (Figure 2). Mouse lungs ventilated with LVt for 4 hours presented normal micromorphology. In contrast, HVt ventilation resulted in leukocyte infiltration, edema and alveolar wall thickening consistent with acute lung injury (Figure 2A). The latter is reflected in the increased lung injury values scored for the HVt untreated group (Figure 2B). Simvastatin minimized histological changes in HVt-treated lungs, thus keeping lung injury scores close to baseline.

### Airspace inflammation is attenuated by simvastatin

**Figure 3:**
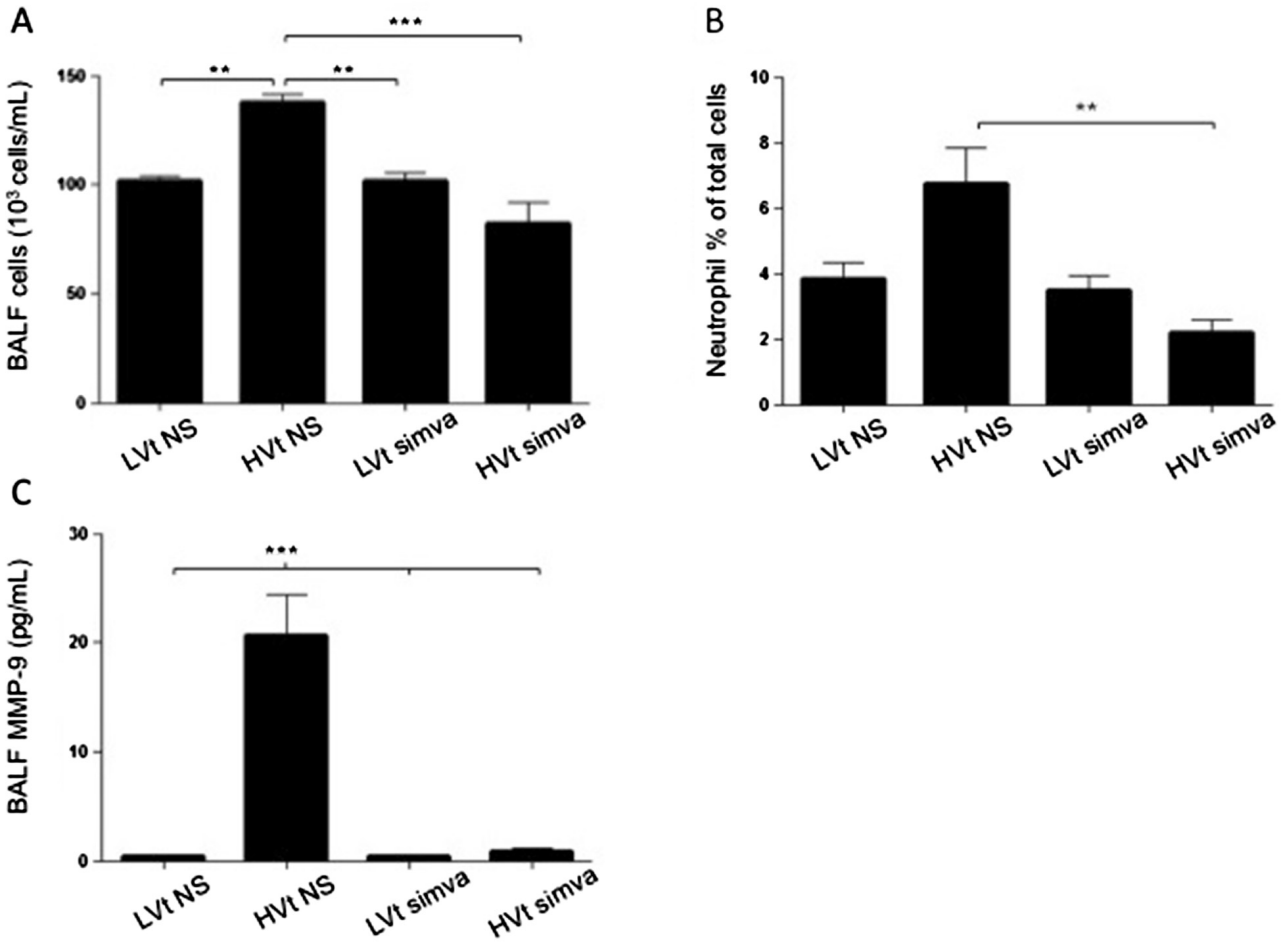
**Effects of simvastatin treatment on airspace inflammation. (A)** Total cell counts in broncho-alveolar lavage fluid (BALF) after 4 hours of mechanical ventilation. **(B)** Neutrophil fraction expressed as percent of total cells in BALF. **(C)** Quantification of matrix metalloproteinases −9 by ELISA in BALF samples of ventilated mice. (LVt: low tidal volume; HVt: high tidal volume; NS: normal saline; Simva: Simvastatin; *denotes p < 0.05; **p < 0.01 and ***p < 0.001).

To assess the lung’s inflammatory response to stretch we measured BALF cell count, differential and levels of matrix metaloprotease-9, which is secreted primarily by neutrophils (Figure 3). We found a modest but significant increase in airspace total cell number after 4 hours of injurious ventilation in placebo-treated mice compared to LVt –ventilated animals. In contrast, BALF total cell numbers did not rise in simvastatin pre-treated mice subjected to HVt (Figure 3A). Neutrophils in BALF behaved in a manner similar to total cell counts (Figure 3B). We then performed ELISA to measure levels of MMP-9, a neutrophil-derived protease known to be involved in murine and human lung injury. Ventilation with HVt induced an approximately 60-fold increase in BALF MMP-9 levels (Figure 3C), which was partly prevented by simvastatin. Notably, plasma MMP-9 levels did not rise in response to HVt (data not shown).

### Systemic inflammation is attenuated by simvastatin

**Figure 4:**
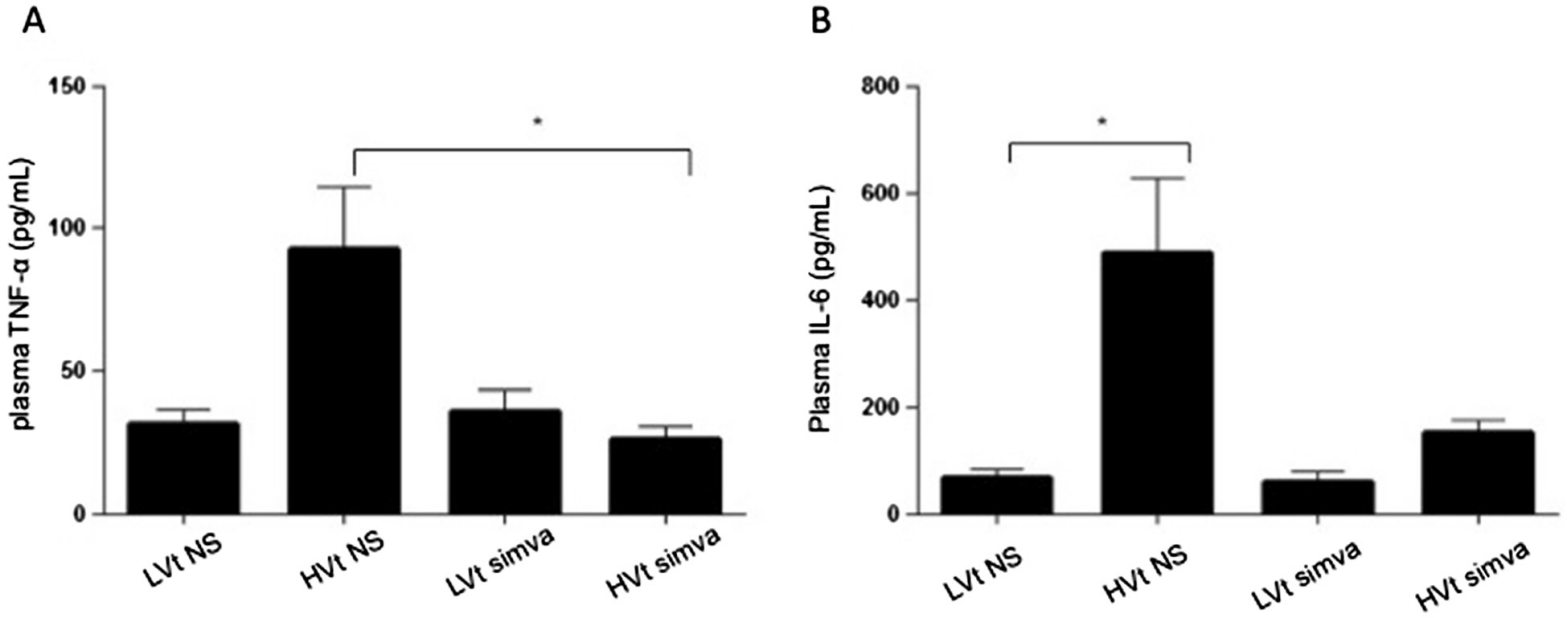
**Proinflammatory cytokines in systemic circulation of ventilated mice.** Levels of TNF-α **(A)** and IL-6 **(B)** were evaluated via ELISA in plasma of mice subjected to low tidal volume (LVt) or high tidal volume (HVt) mechanical ventilation for four hours. (NS: normal saline; Simva: Simvastatin; *denotes p < 0.05; **p < 0.01 and ***p < 0.001).

To assess for potential systemic effects of our model as a marker of severity, we measured plasma levels of inflammatory cytokines TNF-α and IL-6 by ELISA (Figure 4A,B). High-stretch ventilation led to a 3-fold increase in plasma TNF-α level and a 7-fold increase in plasma IL-6 level compared to LVt. Pre-treatment with simvastatin suppressed systemic inflammation and kept the levels of both cytokines close to the control group.

### Simvastatin effects on vascular integrity

**Figure 5:**
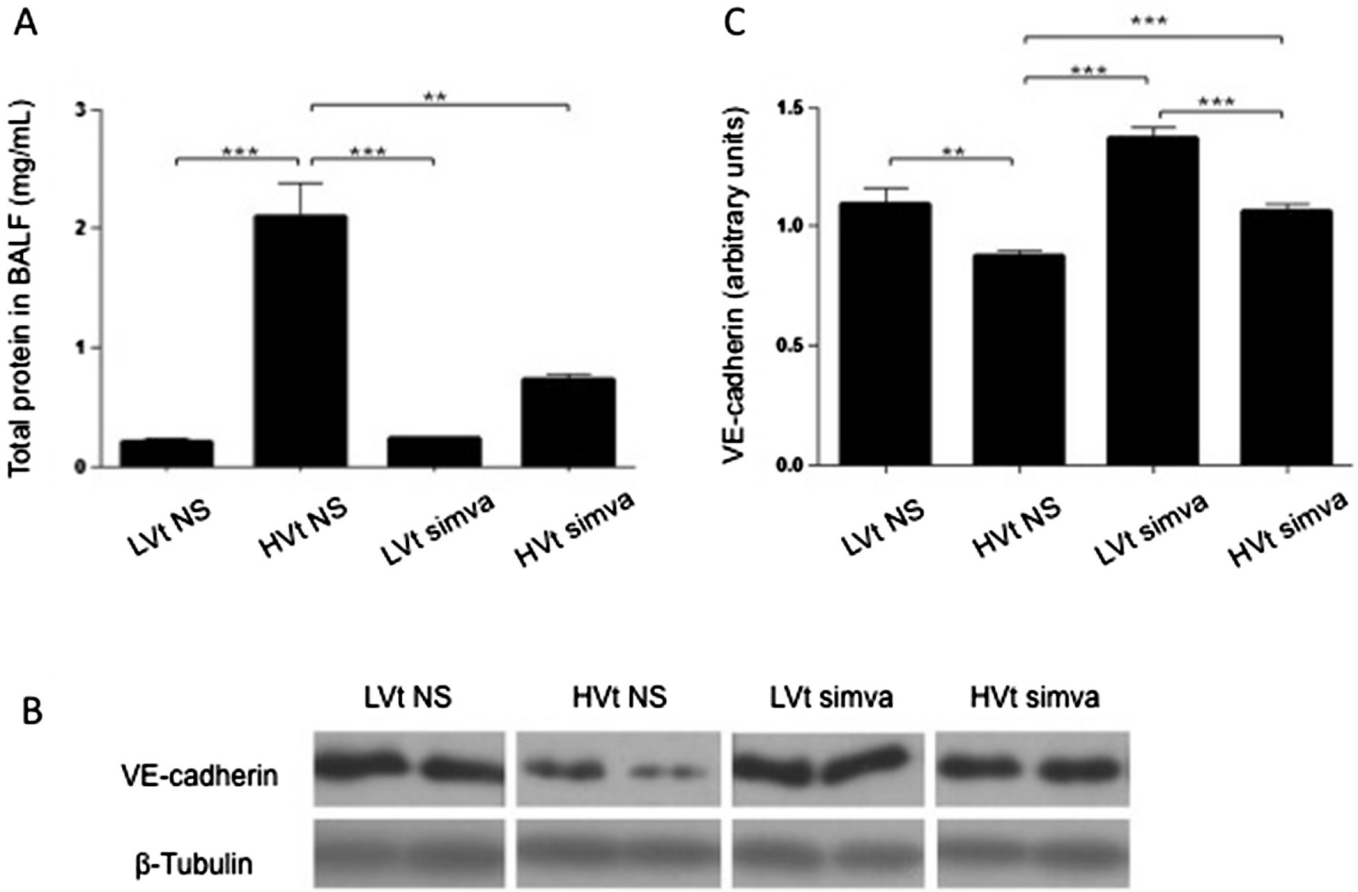
**Effect of simvastatin on endothelial dysfunction in experimental ventilator lung inury. (A)** Total protein content in BALF was used as a marker of endothelial barrier disruption by high-tidal-volume ventilation for four hours. **(B)** Representative immunoblotting of Vascular Endothelial (VE)-Cadherin, the structural protein of endothelial adherens junctions, in mouse lung tissue. **(C)** Quantification of intensity ratio of VE-Cadherin in reference to β-tubulin in lung samples. (LVt: low tidal volume; HVt: high tidal volume; NS: normal saline; Simva: Simvastatin; *denotes p < 0.05; **p < 0.01 and ***p < 0.001).

Pulmonary edema formation in ARDS relies on increased microvascular permeability as a result of disruption of inter-endothelial junctions comprising tight and adherens junctions. We determined BALF total protein concentration to probe the structural integrity of the endothelial barrier in the VILI model (Figure 5A). We observed excessive protein accumulation in the alveolar space in mice subjected to HVt ventilation. Simvastatin pre-treatment resulted in a 3-fold decrease in BALF protein compared to the sham-treated HVt group, without influencing BALF protein in LVt animals. We then examined protein expression of VE-cadherin, the structural protein of adherens junctions (Figure 5B,C). Ventilation with HVt led to a significant decrease in VE-cadherin levels in lung homogenates by immunoblotting, with or without the presence of simvastatin.

## Discussion

We report that high-dose simvastatin pre-treatment can prevent lung injury and rescue pulmonary endothelial dysfunction in a mouse model of mechanical ventilation with supra-normal inflation volume. Excessive stretch can be imposed on the alveolo-capillary membrane during a tidal inflation when the applied Vt is disproportionate to the aerated lung volume. Despite widespread use of “lung protective” ventilation with low Vt, this situation can still take place in the setting of collapsed lung and high PEEP in ARDS patients [2]. Since low Vt ventilation carries the risk of hypercapnea, its use may be contraindicated altogether in pregnancy or intracranial hypertension. In several cases of refractory hypoxemia despite conventional therapy including prone positioning, extracorporeal oxygenation may be of benefit, although this treatment is feasible in a highly selected patient group and available in very few reference centers only. Therefore, when one considers the potential impact of VILI on morbidity and mortality, the need for a pharmacologic treatment becomes obvious.

Simvastatin, a 3-hydroxy-3-methylglutaryl-coenzyme A reductase inhibitor, has been shown to have a number of endothelial-protective and immunomodulating effects and has thus been proposed as an interesting investigational agent for ARDS. Simvastatin pre-treatment in experimental animals has been beneficial in various lung injury models including endotoxin administration, radiation lung injury, allergic asthma and bleomycin-induced fibrosis [6,7,22-24]. Although a number of putative mechanisms acting both on the endothelium and the innate immune system have been suggested, few studies have put these to rigorous testing. Using a model of multisystem inflammation, Rinaldi *et al*. reported that simvastatin pre-treatment in mice induces peroxisome proliferator-activated receptor alpha (PPAR-alpha), which suppresses transcription of pro-inflammatory genes [25]. The relevance of this pathway was demonstrated by the fact that simvastatin protection was lost in PPAR-alpha gene-disrupted mice. Anti-inflammatory effects of statins were also documented by Zhang *et al*. [26], who showed a reduction in neutrophil infiltration to the lung in response to challenge with streptococcal antigen M1 due to decreased production of CXC chemokines. Focusing on the endothelium, Chen *et al*. found that simvastatin induced integrin β4 expression, which could have an effect on endothelial barrier stability and signaling [27]. The plethora of desirable actions of statins spanning the endothelium and possibly epithelium of the lung along with the innate immune system is what makes these drugs so interesting in the treatment of ARDS.

In our model, ventilation of mice with 25 mL/Kg leads to a fairly rapid and time-dependent increase in pulmonary vascular permeability coupled to increased lung elastance resulting from fluid accumulation and surfactant depletion. There is also a moderate inflammatory response in the alveolar space, manifest as BALF total cell and neutrophil accumulation. Neutrophils are phagocytic but also secretory cells and their products, including oxidants and protelolytic enzymes, cause tissue destruction. In this context, we detected a very robust increase of MMP-9 in this model. The main role of MMPs is the degradation of extracellular matrix components (e.g. collagen), and they are involved in both physiological (embryogenesis) and pathological processes (ARDS [28], bronchial asthma [29]). MMP-9 is produced in several types of inflammatory cells in response to cyclic mechanical stretch, including neutrophils and alveolar macrophages [30,31]. Moreover, inhibition of MMP-9 dampens neutrophil-mediated inflammation [32] and protects against the development of VILI. These findings imply a role of MMP-9 in fostering microvascular membrane failure in ARDS. The dramatic reduction in MMP-9 seen with simvastatin pre-treatment attests to the unambiguous protective effect of the drug in this particular disorder. Since no rise in systemic levels was found in our mice, passive leak of MMP-9 from the bloodstream into the alveolar space is improbable and argues for local generation. Finally, the rise in plasma cytokines indicates a systemic component to the inflammatory response as well, which was also abrogated by simvastatin.

Microvascular barrier failure is documented by the presence of protein-enriched BALF in mice ventilated with HVt. Permeability edema is widely accepted to arise from weakening of inter-endothelial and inter-epithelial adhesion structures, comprising adherens and tight junctions. Disruption of cell-matrix contact and basal membrane integrity is probably also contributory. However, how this process unfolds from a mechanistic standpoint is incompletely understood. In this study, we are among the first to provide evidence for a reduction in levels of VE-cadherin by injurious ventilation. Since VE-cadherin is the main structural component of adherens junctions [33], loss of VE-cadherin is expected to compromise endothelial continuity and promote vascular leak. The mechanism behind the decrement in VE-cadherin could have implications on our understanding of permeability edema. Potential explanations include repression of VE-cadherin expression, protein breakdown by proteases or enhanced turnover.

As described in our previous study in isolated lungs [4], pretreatment with statins had a dramatic effect on lung injury from high-stretch ventilation with almost complete protection. This seems to be a class-specific effect, as it was observed with both atorvastatin in the ex-vivo rabbit model and simvastatin in the in vivo mouse model. Similar results were presented in a prior study by Mueller et al. using a mouse model of ventilation with a Vt of 12 mL/Kg. The authors reported that mechanical ventilation induced simvastatin-sensitive microstructural changes on the pulmonary endothelium, consisting of reduction of the number of caveolae, cell-membrane invaginations responsible for transendothelial transport of macromolecules and cell-signaling processes. Our present study adds to the previous observation using a VILI model of at least twice the severity. The almost complete protection afforded to animals by the study agent underscores the potential role of statins as a useful adjunct to the treatment of ARDS. Why is it then that human trials have been met with such dismal failure? Although we cannot be sure, we may speculate that one possibility could be that the doses used are not sufficiently high. Although no major undesirable effects have been demonstrated with the doses used in clinical trials, one would wonder whether higher doses could provide a more robust effect, if tolerated. Changing the route of administration could also be of value, e.g. choosing inhaled vs. enteral. Finally, one major drawback of all clinical research in ARDS is its etiological variability, which precludes the formation of homogeneous patient samples and thus therapeutic effects of drugs on specific patient populations may be missed.

## Conclusion

In conclusion, we show that high-dose simvastatin pretreatment can preserve lung function by preventing inflammation and microvascular dysfunction in a mouse model of severe ventilator injury. Statins may be clinically useful in minimizing the noxious effects of mechanical ventilation in patients with acute lung injury and the acute respiratory distress syndrome.
